# Gender-specific motivation for training in sexual history

**DOI:** 10.3389/fmed.2025.1734934

**Published:** 2026-01-26

**Authors:** Nadja Platzer, Janine Utz, Maximilian Bailer, Nina Triebner, Gian-Marco Kersten, Johannes Kornhuber, Philipp Spitzer

**Affiliations:** 1 Department of Psychiatry and Psychotherapy, Friedrich-Alexander-Universität Erlangen-Nürnberg, Erlangen, Germany; 2 Department of Child and Adolescent Psychiatry, Aalborg University Hospital, Aalborg, Denmark

**Keywords:** expectancy-value theory, gender differences, medical students, psychiatry, self-determination-theory, sexual history, student motivation

## Abstract

**Objective:**

Although sexual history taking is an essential component of a comprehensive medical history, it is often omitted in practice. To ensure competent assessment, this skill should be taught during medical school. However, interest in the topic—particularly among male students—remains limited. This study therefore aimed (1) to examine the impact of gender on students’ motivation to learn sexual history taking and (2) to identify gender-independent factors. The findings are intended to inform teaching innovations that foster motivation in all students and, ultimately, improve the quality of patient care.

**Methods:**

A cross-sectional online survey was conducted in the winter semester 2023/2024 among fifth- to eighth-semester medical students at Friedrich-Alexander University Erlangen-Nuremberg. The questionnaire comprised the Learning Self-Regulation Questionnaire (SRQ-L) and 11 self-developed items addressing potential influences on students’ motivation to learn sexual history taking. Data were analyzed using *t* tests, ANOVA, regression, and mediation analyses.

**Results:**

A total of 318 students participated (86 male, 232 female). Perceived relevance of the topic and the psychiatric clinic as the organizer of the elective course were significant predictors of motivation. Female students showed higher autonomous regulation than males [*t* (118.68) = -3.48, *p* < 0.001, d = 0.51] and rated the topic as more relevant [*t* (121.39) = -3.86, *p* < 0.001, *d* = 0.56]. Mediation analysis revealed that the gender effect on autonomous regulation was fully mediated by perceived relevance [indirect effect ab = 0.3231, 95% CI (0.160, 0.4996)].

**Conclusion:**

Gender differences in motivation to attend a voluntary seminar on sexual history taking are explained by perceived relevance rather than biological sex. Relevance plays a central role in fostering autonomous motivation. Therefore, curricular strategies should emphasize the importance of sexual history taking to increase engagement across all genders. Integrating this topic into the compulsory curriculum may compensate for initial gender disparities and contribute to long-term improvements in medical interviewing and patient care.

## 1 Introduction

Sexual health is fundamentally important to wellbeing (1), which means that sexual history also plays a key role in good medical care. Problems with sexual health are highly important in the context of patient care, which can be attributed to the high prevalence of these problems. A substantial proportion of patients—approximately one-third of men (33.4%) and nearly half of women (45.7%)—are affected by sexual problems (2). As part of a comprehensive sexual history and the subsequent examination, it is possible to diagnose not only sexual dysfunctions—whether idiopathic or drug induced—but also sexually transmitted infections (STIs) and psychosexual stress (3). A substantial proportion of physicians, specifically 90%, consider information pertaining to sexual health to be pertinent. However, only half of these physicians actively inquire about this subject. A salient issue is the dearth of knowledge concerning the management of sexual dysfunction (1). According to previous studies, the vast majority of patients, specifically 90%, express a desire to be asked about sexual health by their doctor (4). However, it has been demonstrated that only 25% of German patients initiate a discussion on this topic (5). A considerable disparity between the needs of patients and the willingness of medical staff to engage in discourse on this subject has been identified. Many medical professionals report experiencing a sense of personal discomfort when discussing issues pertaining to human sexuality. Conversely, patients are increasingly forthcoming about these matters and expect—and in many cases demand—that their doctors should be proficient in this domain (6). Furthermore, the enhancement of sexual history taking would facilitate an optimized diagnosis and therapy, thereby promoting an improvement in the therapeutic relationship (6).

The integration of sexual history into the curriculum is not a novel concept. Nevertheless, to date, little evidence of this implementation has been presented. However, there is a clear need for teaching and practice among students (1). In accordance with the current subject catalog 2 of the IMPP (Institute for Medical and Pharmaceutical Examination Questions), which forms the basis for German state examinations in medicine, taking a sexual history is listed as an expected competency by the time of the second state examination (see GK2 D 1.4.7). In accordance with the prevailing German National Competence-Based Learning Objectives Catalog for Medicine (NKLM), the documentation of a sexual history is categorized at level 3b at the end of the degree program, which is consistent with the highest level of competence as defined by the NKLM (see NKLM VIII.2-02.4.7) (7). However, the implementation of these requirements has been found to be largely inadequate. This situation is evidenced by a study from Hamburg-Eppendorf, in which 49–84.1% of students evaluated the current teaching program for sexual medicine as inadequate (1). A review of the prevailing medical curricula in Germany revealed that the subject of sexuality and communication regarding sexuality is allocated a mere 10 h of instruction (1). A recent study from Finland lends further support to the aforementioned research by Turner et al., indicating that medical students report a deficiency in their knowledge regarding the assessment of sexuality and the treatment of sexual problems (8).

Consequently, the onus to expand their understanding of the subjects of sexual history and sexual medicine appears to be on students themselves. Notably, certain universities provide this opportunity in the form of elective courses. At Julius-Maximilians University of Würzburg, for instance, a course on sexual history is offered and is taught by students in a peer-to-peer format. Students evaluated this course as being of significant value and as a highly beneficial contribution to the expansion of their skills and knowledge in the area of sexual history taking. Consequently, the course has been incorporated into the Würzburg curriculum for the 9th semester as a permanent component since the 2022/23 winter semester (9). Friedrich-Alexander University of Erlangen-Nuremberg also offers an elective course on the subject of learning sexual history taking, titled “Let’s talk about sex—Learning sexual history taking in practice”. Furthermore, the acceptance rate of this offer is notably high among students. However, it is notable that a significantly high proportion of participants in this course—in some semesters, over 90%—are female. The reasons for the considerable underrepresentation of male students remain unclear, and it engenders a particular issue. The potential disparities that may emerge regarding communication skills and variations in quality of care are influenced by the gender of the treating physician. It has previously been demonstrated that gender is not the only significant factor in patient satisfaction with communication. As demonstrated by a Swiss study, it is not gender itself that is decisive but, rather, gender-specific factors such as communication style. The findings of this study indicate that patients evaluated female gynecologists’ communication skills more favorably than they did those of their male counterparts. This phenomenon can be attributed to the more patient-centered communication style of female doctors (10). The findings suggest the existence of gender-specific factors, such as communication style, in relation to sexual history taking. These factors may influence the quality of sexual history taking and, consequently, patient satisfaction and compliance. In our estimation, these gender-specific factors could be counterbalanced by the implementation of appealing teaching methods, with a particular emphasis on enhancing the motivation of male students. It is therefore presumed that a gender-equitable teaching concept could lead to a more balanced group of participants and thus contribute to ensuring quality of care.

To address this research gap, an observational cross-sectional study was conducted at Friedrich-Alexander University of Erlangen-Nuremberg. The objective of this study was to ascertain gender-specific motivational factors and potential barriers to participation in the elective course on learning how to take a sexual history. Consequently, the objective is to optimize the course offering with regard to the identified motivational factors, thereby achieving a long-term improvement in the future medical care of patients.

## 2 Materials and methods

This cross-sectional study was conducted via an online questionnaire during the clinical semesters of the medicine degree program at Friedrich-Alexander University of Erlangen-Nuremberg. The data were collected anonymously, and a note to this effect was included at the beginning of the questionnaire. Prior to commencing the questionnaire, participants were required to provide consent for the utilization of the collected data. Participation in the survey was voluntary, and students were not subject to any positive or negative consequences for their involvement. The survey was conducted in accordance with the principles of the Declaration of Helsinki. This study was submitted to the Institutional Review Board (ethical committee) of the Friedrich-Alexander University of Erlangen-Nuremberg and received a designation of exempt according to §15 BO (professional code of conduct for the physicians of Bavaria).

### 2.1 Participants

The survey was administered at the beginning of the 2023/2024 winter semester on three designated dates (October 23rd, 24th, and 25th, 2023). During a lecture in the 5th–8th semesters (1st–4th clinical semesters), a QR code was presented that could be scanned by the students in attendance. This code directed the students to the SoSci-Survey platform. The QR code was presented either at the commencement or at the conclusion of the lecture. Furthermore, on November 6th, 2023, the survey link was disseminated via the WhatsApp communication platform to different groups ranging from the 5th to the 8th semesters. The survey period was from October 23rd, 2023, to November 17th, 2023. Furthermore, participants in the elective course, entitled “Let’s talk about sex—Learning sexual history taking in practice”, were surveyed on three occasions (April 28th, 2023; December 15th, 2023; and May 10th, 2024). The questionnaire was written in German, which is why knowledge of the German language was included as a prerequisite for participation in the survey. The sole inclusion criterion for the study was enrollment in the clinical section of the medicine degree program at Friedrich-Alexander University of Erlangen-Nuremberg.

### 2.2 Questionnaire

The “Learning Self-Regulation Questionnaire” (SRQ-L), which comprises 14 questions, was utilized in this study. The text was adapted to the subject of sexual history and translated into German. The internal consistency of the questionnaire with regard to autonomous regulation (seven questions) can be rated as good (*Cronbach’s alpha* = 0.846). However, the internal consistency of the controlled regulation subscale (seven questions) can be considered questionable (*Cronbach’s alpha* = 0.654). Furthermore, the questionnaire incorporated a series of eleven self-formulated inquiries that aimed to quantify the influence of the framework conditions and individual factors of the elective course. The framework conditions of the elective course included the psychiatric clinic as the organizer, the course’s scheduling on the weekend, and the preference for gender-segregated groups. The personal influencing factors examined included the individual’s sense of competence, the relevance attributed to sexual history, comfort in talking about sexuality in a student/professional context, comfort as a patient during a sexual history interview, the number of sexual history interviews already experienced as a patient, and the assessment of patients’ need to be asked about their sexual health in a general practitioner context. Personal criteria can be applied to medical students, regardless of their academic institution. Moreover, expectations regarding the elective subject and the targeted medical specialty were collected as free-text responses. At the commencement of the questionnaire, demographic data pertaining to gender and the semester of study were collected.

### 2.3 Data analysis

Statistical analysis was performed with the assistance of SPSS version 29.0.1.0 software (IBM SPSS Statistics). Figures were created via PRISM 10. Group differences were examined via independent sample *t* test and ANOVA. Effect size is represented via Cohen’s *d*. The Spearman correlation coefficient *r* is used as a measure of correlation. Values of *p* < 0.05 are considered significant. The regression coefficient β is used for a dimensionless comparison of the different variables. Variance equality was tested, and Welch’s correction was performed where necessary. Mediation analyses were performed via the PROCESS v4.2 macro for SPSS (Andrew F. Hayes).

## 3 Results

### 3.1 Descriptive statistics of the collected data

The data analysis included 500 questionnaires, of which 318 were fully completed, corresponding to an approximate effective response rate of 44% of the 725 students enrolled in the fifth to eighth semesters. Among the respondents, 86 identified as male, constituting 27% of the sample, whereas 232 identified as female, accounting for 73% of the sample. This distribution is consistent with the gender distribution observed in the medical degree program of Friedrich-Alexander University of Erlangen-Nuremberg. One participant indicated “diverse,” but their response was excluded from the evaluation to preserve the confidentiality of the data. The survey participants were predominantly in their fifth to eighth semesters at the time of the survey, with 96.3% (*n* = 307) of the respondents falling into this category. The remaining respondents (3.7%) were not actively engaged in the survey but were exposed to it incidentally. The proportion of participants in the elective course was 12.3% (*n* = 39). Table 1 presents a comprehensive array of demographic data.

**TABLE 1:** Demographic data of the respondents.

Respondents		Male	Female	Overall
Total		86	232	318
Semester	5	25	47	72
	6	10	26	46
	7	21	45	66
	8	25	85	110
	9	2	4	6
	10	1	0	1
	11	0	2	2
	12	0	2	2
Participation in the elective course	Yes	6	33	39
	No	80	199	279

### 3.2 Men are less motivated to learn sexual history than women

**FIGURE 1:**
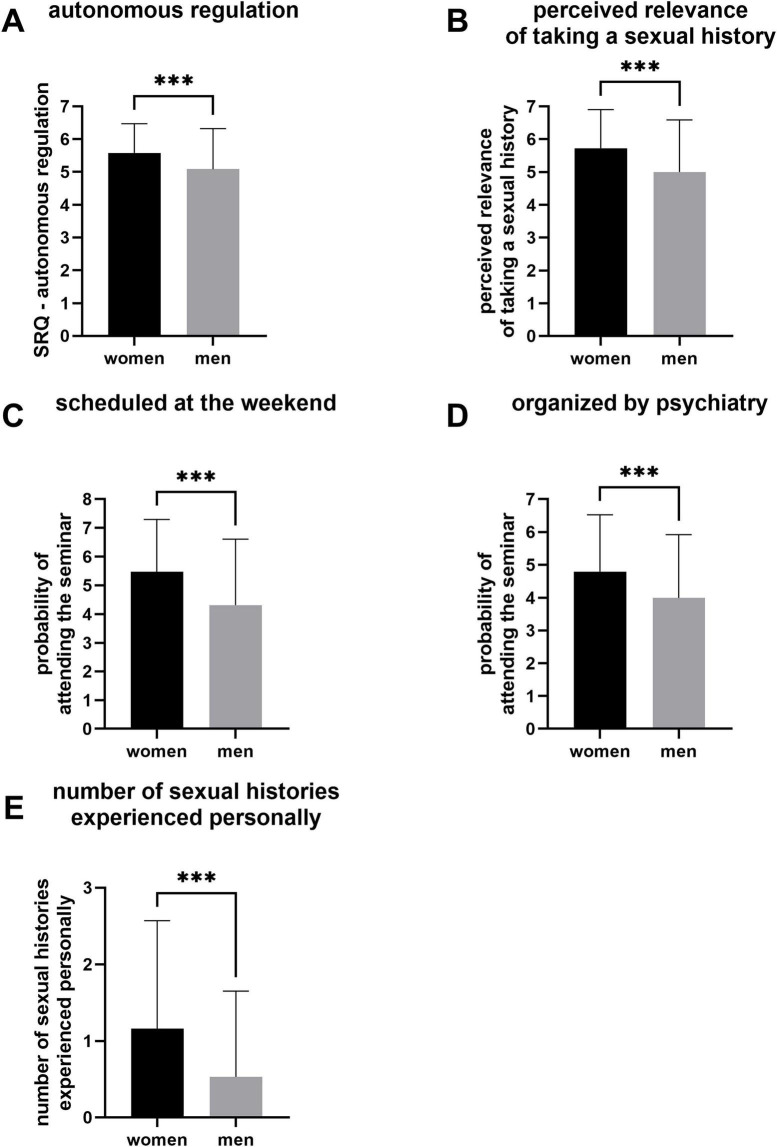
Significant results of independent sample ***t*** tests showing the discrepancy between female and male medical students in **(A)** autonomous regulation, **(B)** the elective course being organized by psychiatry, **(C)** the elective course being scheduled at the weekend, **(D)** perceived relevance of taking a sexual history, **(E)** number of sexual histories experienced personally. ***p < 0.001.

Independent sample *t* tests were performed to conduct group comparisons between female and male students. The evaluation demonstrated that compared with their female counterparts, male students exhibited lower autonomous regulation to engage in sexual history taking [*t*(118.68) = -3.48, *p* < 0.001, *d* = 0.51] (see Figure 1A). Furthermore, a significant discrepancy in the ratings of sexual history relevance among male and female students was observed, with female students assigning a higher rating than their male counterparts did [*t*(121.39) = -3.86, *p* < 0.001, *d* = 0.56] (see Figure 1B). In contrast to male respondents, female respondents were more likely to enroll in a weekend course [*t*(126.79) = -4.20, *p* < 0.001, *d* = .59] (see Figure 1C). Furthermore, male students indicated a reduced propensity to enroll in elective courses at the psychiatric clinic [*t*(316) = -3.56, *p* < 0.001, *d* = 0.45] (see Figure 1D). A significant discrepancy in the number of sexual histories taken reported by the respondents was observed, whereas women were found to have experienced sexual histories more frequently than men [*t*(178.20) = -4.05, *p* < 0.001, *d* = 0.48] (Figure 1E). The variables *identification with the biological gender*, *extrinsic motivation*, *preference for gender-segregated groups*, *perceived competence in taking a sexual history*, *comfort level in discussing sexuality within a student/professional context*, *comfort level as a patient during sexual history taking*, and the *assessment of the necessity for patients to be asked about their sexual health by their primary care physician* did not reveal any significant group differences.

### 3.3 Attributed relevance as the most important factor influencing autonomous regulation

Multiple linear regression with stepwise inclusion was performed to quantify the influence of the surveyed variables on *autonomous regulation*. The variables included in the study were as follows: *the relevance attributed to the sexual history*, *the psychiatric clinic as the organizer of the elective course*, *the implementation of the elective course in gender-segregated groups*, *the comfort level in talking about sexuality in a student/professional context*, and *the semester in which the respondents were enrolled*. *Autonomous regulation* was selected as the dependent variable because it is the most significant predictor of participation [*r*(316) = 0.535, *p* < 0.001]. The model, which incorporated *the relevance attributed to sexual history* as the sole influential variable, exhibited a high variance explained of *R*^2^ = 0.355 (corrected *R*^2^ = 0.353). The overall model indicated a high level of variance explained (*R*^2^ = 0.443; corrected *R*^2^ = 0.433). In contrast to the initial hypothesis, gender was no longer incorporated into any of the models. In a linear regression model with *gender* as the sole independent variable, the result was nevertheless significant [*F*(1, 317) = 16.312, *p* < 0.001], indicating a modest variance explained (*R*^2^ = 0.049; corrected *R*^2^ = 0.046). The results of the individual regressions with the influencing variables from the overall model as independent variables are shown in Table 2.

**TABLE 2:** Results of individual regressions with the influencing variables from the overall model including *perceived relevance of sexual history, the psychiatric clinic as the organizer of the voluntary course on sexual history, comfort in talking about sexuality in a student/professional context, the semester in which the students are enrolled, the preference for the course to be held in gender-segregated groups* as independent variables.

Independent variable	*R* ^2^	Regression coefficient *B*	Standard deviation	Standardized coefficient β	95% CI [minimum, maximum]
Relevance	0.362	0.458	0.034	0.602	[0.391, 0.525]
Psychiatry	0.141	0.210	0.029	0.376	[0.153, 0.276]
Comfort in talking about sexuality in a student/professional context	0.044	0.160	0.042	0.209	[0.077, 0.243]
Semester	0.009	0.072	0.043	0.095	[-0.013, 0.156]
Preference for gender-segregated groups	0.065	0.159	0.034	0.255	[0.092, 0.226]

### 3.4 Attributed relevance explains differences in autonomous regulation between the genders

**FIGURE 2:**
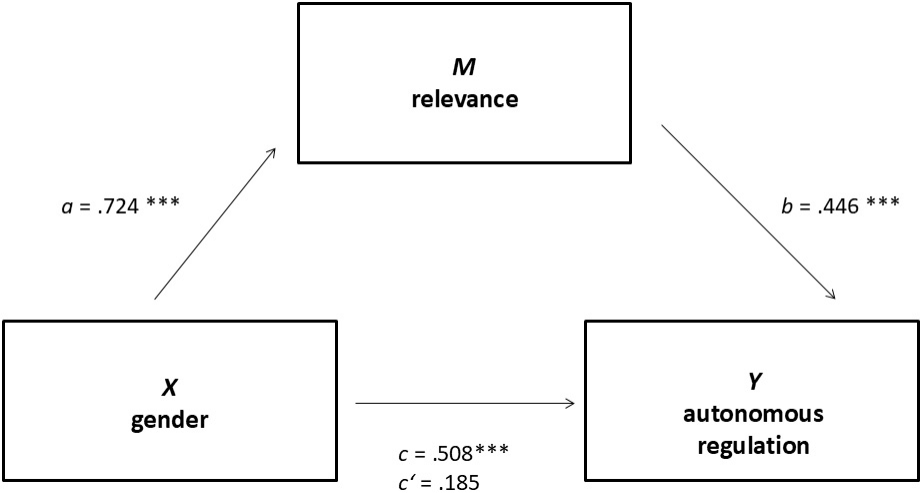
Complete mediation between independent variable gender (X) and dependent variable autonomous regulation (Y) with relevance as mediator (M). ***p < 0.001.

**FIGURE 3:**
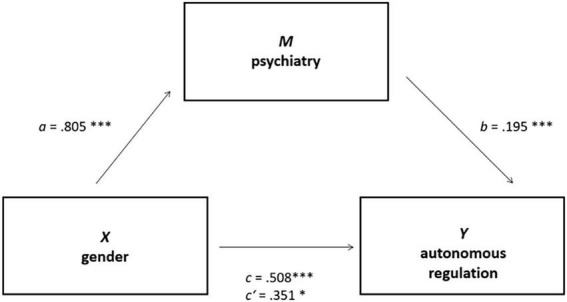
Partial mediation between independent variable gender (X) and dependent variable autonomous regulation (Y) with the psychiatry as mediator (M). *p < 0.05, ***p < 0.001.

Mediation analyses were performed testing all variables shown to be significant predictors in the regressions models as potential mediators one by one. The independent variable in this analysis was *gender*, while the dependent variable was *autonomous regulation*. The sole instance of complete mediation was observed with *relevance* as the mediator (see Figure 2), with an indirect effect of *ab* = 0.323, 95% CI [0.160, 0.500]. This mediation demonstrates that female medical students exhibit higher levels of autonomous regulation, attributable to a heightened perception of the relevance of sexual history taking. The mediation analysis with the mediator *psychiatric clinic as the organizer of the elective course* indicated partial mediation, with an indirect effect *ab* = 0.157, [0.059, 0.277] (see Figure 3). This suggests that the psychiatric clinic as the organizer is a key factor in the decision to enroll in the voluntary course on the subject of sexual history taking. The remaining mediation analyses did not demonstrate any significant mediations. Comprehensive data are presented in Table 3.

**TABLE 3:** Results of individual mediations performed with *gender* as the independent and *autonomous regulation* as dependent variable.

Mediator	Total effect *c*	Indirect effect *ab*	Direct effect *c’*	95% CI [minimum, maximum]
Relevance	0.5079 ***	0.323	0.185 ***	[0.160 ***, 0.4996]
Psychiatry	0.508 ***	0.157	0.351*	[0.805 ***, 0.195 ***]
Comfort in talking about sexuality in a student/professional context	0.508 ***	–0.046	0.554 ***	[-0.262, 0.013]
Semester	0.508 ***	0.014	0.494 ***	[0.247, 0.058]
Preference for gender-segregated groups	0.508 ***	0.047	0.461 **	[0.316, 0.150 ***]

*p < 0.05,

**p < 0.01,

***p < 0.0001.

### 3.5 Influence of the targeted medical specialty

**FIGURE 4:**
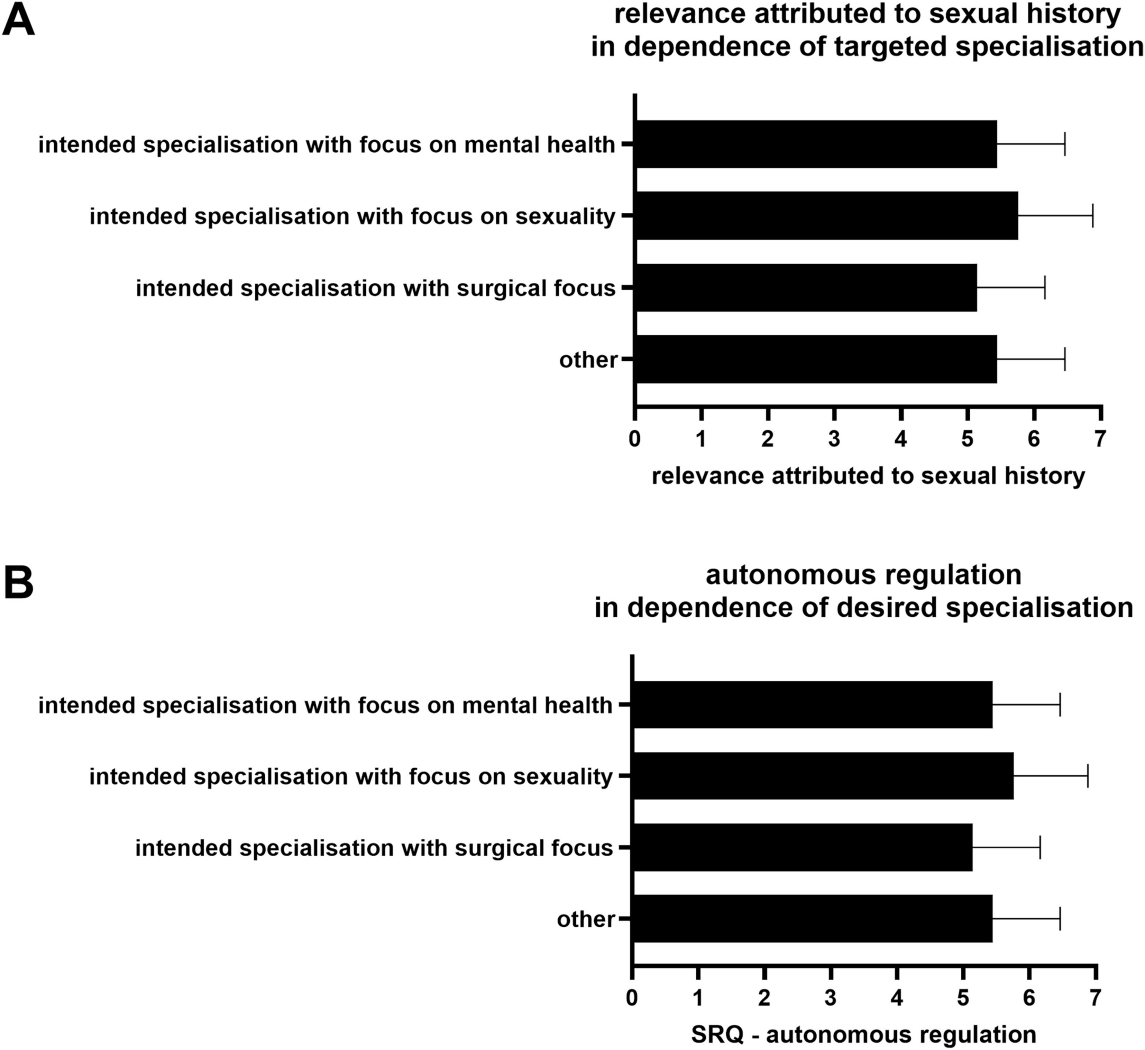
**(A)** perceived relevance of taking a sexual history, depending on the targeted medical specialty **(B)** Autonomous regulation to learn sexual history taking, depending on the targeted medical specialty.

The study also examined the relationship between the targeted medical specialty and the relevance attached to sexual history (see Figure 4A). For this purpose, the targeted medical specialties stated as free text were initially grouped accordingly. Owing to the insufficient number of responses received from each specialty, conducting a comprehensive differentiation of the respective areas of expertise was not feasible. Instead, the following four groups were defined: fields related to surgery (surgical disciplines, anesthesia, and orthopedics), fields related to gender (gynecology, urology, and dermatology), psychiatric fields (psychiatry, child and adolescent psychiatry, and psychosomatics), and “other fields” (all fields not mentioned above). In the context of this study, students who indicated surgery as their future career goal demonstrated the lowest ratings of sexual history relevance among the groups examined [*M* = 5.06, 95% CI (4.70, 5.42)]. Students who intend to pursue specializations in psychiatric subjects [*M* = 6.36, (5.91, 6.82)] or gender-related subjects [*M* = 6.00, (5.66, 6.34)] assigned a higher level of relevance of sexual history (*p* < 0.001). Subsequent group comparisons pertaining to disparate assessments of the relevance of sexual history proved to be nonsignificant.

A similar pattern was observed with respect to autonomous regulation (see Figure 4B): Students who aspire to specialize in a surgical discipline exhibited notably lower levels of autonomous regulation to acquire knowledge about sexual history taking [*M* = 5.139, 95% CI (4.891, 5.387)]. This observation stood in clear contrast to the findings regarding their peers pursuing medical specialties related to gender [*M* = 5.762, (5.436, 6.088)] or psychiatry [*M* = 6.00, (5.434, 6.566)], *p* = 0.003. Further analyses revealed no substantial disparities between the groups in terms of autonomous regulation.

## 4 Discussion

The present study investigated the hypothesis that the motivation to learn sexual history taking is contingent on the student’s gender. The study also sought to identify gender-independent factors that could influence this motivation. The results demonstrated that motivation levels among female medical students to learn how to take a sexual history were significantly higher than the levels among male medical students. This phenomenon can be attributed to the heightened relevance that women ascribe to sexual history taking. Consequently, the motivation to attend a seminar on learning sexual history taking is not influenced by gender but rather by the relevance attributed to sexual history. A further salient finding of this study is that elective courses in psychiatry are less likely to be taken by men than by women.

The findings of this study demonstrate that compared with male subjects, female subjects attribute significantly greater relevance to sexual history taking. A similar correlation was also identified in a study by Ludwig et al., which revealed that female participants rated gender-sensitive medicine as more relevant than their male counterparts did, with gender being identified as the only significant influencing factor. Their study posits that other diversity factors, such as having children or a disability, exert no significant influence (11). The majority (62%) of the students surveyed in that study agreed that gender-sensitive medicine is a pertinent field of study. The gender disparity observed in this context is noteworthy: 68% of female students agreed with this statement, whereas only 51% of male students agreed (11). The findings of the present study on sexual history, in conjunction with those of the aforementioned study on gender-sensitive medicine, indicate a substantial discrepancy between the genders. A further distinction between the genders is evident in terms of individuals’ attitudes toward and openness to subjects related to sexuality. Compared with their male counterparts, female medical students exhibit a reduced tendency toward stereotypical attitudes (12). Furthermore, female lecturers demonstrate a heightened level of openness and a greater interest in training events that address subjects pertaining to sexuality (13). These findings align with the hypothesis that women exhibit heightened sensitivity to issues pertaining to sexual health.

Furthermore, research has revealed that men, including those who occupy senior positions within medical schools, regard gender-related issues as significant. However, they evaluate the status of gender-related issues as low and consequently consider them to be irrelevant to medical expertise (14). An ambivalent attitude toward issues of sexuality can therefore be observed among men, which may have a significant impact on the scope and quality of teaching in this area. Furthermore, Risberg et al. hypothesized that increased engagement by men in these subjects could contribute to a greater perception of their relevance (14). The extant evidence thus appears to suggest that structural social factors could influence the perceived relevance of topics in sexual medicine, including sexual history taking. Consequently, students’ motivation to learn how to take a sexual history is also influenced by these structural social factors.

As posited by Burd et al., the discomfort experienced by medical staff themselves constitutes a significant impediment to the effective collection of sexual histories (15). However, the findings of their study could not be substantiated by the results obtained. Both male and female participants reported a sense of confidence in discussing sexuality within the context of a student/professional environment. Nevertheless, the results of the aforementioned study reveal key factors related to the challenges that arise when taking a sexual history in everyday practice. The results of the analysis indicate that medical staff experience elevated levels of discomfort when they conduct sexual history interviews with patients of the opposite sex. In contrast, medical professionals have been observed to perceive patient discomfort as more pronounced when both parties involved are of different sexes (15). This study posits that other influential factors include patient age, such that minors and patients older than 60 in particular cause discomfort among medical staff when taking a sexual history. This problem is significant, as these patient groups are at increased risk of sexual dysfunction (15). To mitigate the discomfort experienced by healthcare professionals, enhanced pedagogical approaches concerning sexual history taking are necessary (15). In particular, there is a need to integrate pediatrics and geriatrics into interdisciplinary curricular teaching on sexual medicine. Another assumption made by Burd et al. is that sexual history is considered of secondary importance. The authors of the study posit that the low response rate of only 59% indicates this point (15). It can be hypothesized that healthcare providers who do not take sexual histories did not participate in the study.

The findings of earlier research indicate that gender *per se* is not the primary influence on the success of sexual history taking. Rather, it is the gender-related communication styles of the individuals involved (10). Compared to their male counterparts, female professionals have been observed to adopt more patient-centered communication styles (10), which have been demonstrated to result in higher patient satisfaction and greater adherence (10). Consequently, it can be deduced that it would be particularly advantageous for male students to acquire the ability to take a sexual history in a practical context, complemented by training in communication skills, to compensate for gender-related disparities in communication skills. Therefore, it is imperative to increase the motivation of male students in particular to engage with the topic of communication. This point is particularly evident in the context of sexual history taking, as this process entails the discussion of sensitive aspects pertaining to health and identity with patients.

In summary, gender differences exist, but these differences are not determined by biological sex. Instead, the pivotal factors pertain to attitudes toward sensitive or gender-related issues and the manner in which communication with patients is conducted, which exhibit gender-based differences.

However, the most significant gender discrepancy pertains to autonomous regulation, which is taking notably more pronounced among female students than among their male counterparts regarding the topic of sexual history. In the context of this study, relevance was identified as the most significant factor in promoting autonomous regulation. This finding can be discussed in conjunction with established theories of motivation formation, such as expectancy-value theory and self-determination theory.

In accordance with expectancy-value theory (16), the determination of whether individuals allocate effort to a task is contingent upon two factors: their expectation of success and their perceived value of the task. If relevance is interpreted as utility value in the sense of expectancy-value theory, this explains why female students, due to ascribing greater relevance to sexual history taking, show greater motivation to acquire knowledge in this field. The extant evidence suggests that the motivation to engage in sexual history taking may be increased by enhancing its perceived relevance. This hypothesis is supported by a substantial body of additional research. For instance, a Norwegian study demonstrated that the intrinsic motivation and effort exhibited by participants in a group that was assigned a task that group members perceived as relevant were greater than in the corresponding comparison group (17). This phenomenon can be explained by the supposition that content is perceived as relevant by learners only if the learning activity aligns with their personal attitudes, interests, and values (17). It has been demonstrated that the recognition of the usefulness of learning content has the capacity to enhance motivation, interest and academic performance (18–20). Accordingly, the perception of the relevance of the content to be learned is of essential importance in this context. Particularly for male students, it is imperative to understand the significance of this subject, as they estimate the relevance of sexual history lower than their female counterparts do. Conversely, an increase in the perception of relevance has the potential to engender an increase in the motivation of male students to engage with the subject of sexual history. This motivation is also significantly lower than that of their female counterparts. In the context of motivation, self-determination theory can also be applied. This theory states that motivation is influenced by three factors: autonomy, relatedness, and competence (21). An emphasis on relevance has been demonstrated to enhance perceptions of autonomy (22), which, in accordance with the principles of self-determination theory, is assumed to positively impact motivation.

In addition to the relevance of taking a sexual history, the psychiatric clinic, as the organizer of the elective course, has proven to be a significant influencing factor. This finding prompts the question of whether gender-based stigmatization of psychiatry has an impact on the motivation to take an elective course on learning how to take a sexual history. International studies have demonstrated that men exhibit a more pronounced stigma toward mental illness (23–26). In contrast, women demonstrate a 1.5 times more positive attitude toward patients with mental illness (27). Furthermore, a correlation can be identified between the stigmatization of mental illness and the choice of medical specialty. A comparative analysis of the attitudes of students preferring surgical disciplines toward psychiatry revealed stronger stigma compared to the attitudes of students preferring other medical disciplines (28). The extant evidence suggests that the targeted medical specialty has an effect on the assessment of the relevance of sexual history because of the associated stigmas toward psychiatry. This hypothesis could be the subject of future studies.

### 4.1 Limitations

Nevertheless, the interpretation of the results of this study is limited by the following factors. It was not possible to determine the precise response rate for the questionnaires because of the variety of methods employed in the distribution of the survey. Consequently, the possibility of selection bias cannot be discounted. It is conceivable that students who were already highly motivated and interested in the topic were particularly likely to participate in the survey. Furthermore, the possibility of gender bias cannot be discounted, as the proportion of female respondents in the survey exceeds the proportion of female students in the clinical section (73% vs. 69%). Given that self-assessment questionnaires were the sole data collection instrument used in this research, the possibility of social desirability bias in the responses cannot be discounted. However, we assume that this influence is minor, as participation in the survey was voluntary and anonymous. The data for the present study were collected at only one university and only in voluntary lectures, which may limit the generalizability of the results of this study. However, given that the questions posed were predominantly of a general nature, independent of the pedagogical concept of Friedrich-Alexander University of Erlangen-Nuremberg, it can be assumed that the results are generalizable. A further limitation is evident in the low Cronbach’s alpha of the questionnaire on controlled regulation. However, given that this variable was not included in the subsequent evaluations, the conclusions of the study are not affected by this issue. Moreover, the self-formulated inquiries used in this study lack standardization, a deficiency that casts doubt on their reliability.

## 5 Conclusion

This study suggests that gender is not the decisive variable in the decision to learn about sexual history; rather, autonomous regulation is the key. This factor, in turn, is significantly influenced by the relevance attached to sexual history. Therefore, motivation could be increased by emphasizing the relevance of sexual history in an interdisciplinary context. This could be implemented either in the various subject areas themselves or in the form of a cross-disciplinary course. In this context, it is essential not only to consider obviously relevant subject areas such as gynecology, urology, dermatology, or psychiatry but also to integrate a broader spectrum of disciplines. The implementation of a curricular subject that addresses the topic of sexual medicine and thus emphasizes the relevance of the subject area, including sexual history, could be one way of overcoming gender differences.

## Data Availability

The raw data supporting the conclusions of this article will be made available by the authors, without undue reservation.
